# Fast and Highly Efficient Adsorption Removal of Toxic Pb(II) by a Reusable Porous Semi-IPN Hydrogel Based on Alginate and Poly(Vinyl Alcohol)

**DOI:** 10.3389/fchem.2021.662482

**Published:** 2021-07-28

**Authors:** Wenbo Wang, Xiangyu Liu, Xue Wang, Li Zong, Yuru Kang, Aiqin Wang

**Affiliations:** ^1^ College of Chemistry and Chemical Engineering, Inner Mongolia University, Hohhot, China; ^2^ Key Laboratory of Clay Mineral Applied Research of Gansu Province, Lanzhou Institute of Chemical Physics, Chinese Academy of Sciences, Lanzhou, China

**Keywords:** alginate, hydrogel, poly(vinyl alcohol), adsorption, lead(II)

## Abstract

A porous semi-interpenetrating network (semi-IPN) hydrogel adsorbent with excellent adsorption properties and removal efficiency towards Pb(II) was prepared by a facile grafting polymerization reaction in aqueous medium using natural biopolymer sodium alginate (SA) as the main chains, sodium acrylate (NaA) as the monomers, and poly(vinyl alcohol) (PVA) as the semi-IPN component. FTIR, TGA and SEM analyses confirm that NaA monomers were grafted onto the macromolecular chains of SA, and PVA chains were interpenetrated and entangled with the crosslinked network. The incorporation of PVA facilitates to form pores on the surface of hydrogel adsorbent. The semi-IPN hydrogel containing 2 wt% of PVA exhibits high adsorption capacity and fast adsorption rate for Pb(II). The best adsorption capacity reaches 784.97 mg/g, and the optimal removal rate reaches 98.39% (adsorbent dosage, 2 g/L). In addition, the incorporation of PVA improved the gel strength of hydrogel, and the storage modulus of hydrogel increased by 19.4% after incorporating 2 wt% of PVA. The increase of gel strength facilitates to improve the reusability of hydrogel. After 5 times of regeneration, the adsorption capacity of SA-*g*-PNaA decreased by 23.2%, while the adsorption capacity of semi-IPN hydrogel only decreased by 10.8%. The adsorption kinetics of the hydrogel in the initial stage (the moment when the adsorbent contacts solution) and the second stage are fitted by segmentation. It is intriguing that the adsorption kinetics fits well with both pseudo-second-order kinetic model and pseudo-first-order model before 60 s, while only fits well with pseudo-second-order adsorption model in the whole adsorption process. The chemical complexing adsorption mainly contribute to the efficient capturing of Pb(II).

## Introduction

In recent years, the rapid development of lead-zinc metallurgy, electroplating, batteries and other industries has discharged a large amount of toxic Pb(II)-containing wastewater, and the resulting water pollution problems become more and more serious, threatening people’s health, survival and development. Presently, a variety of innovative technique has been developed and employed to decontaminate the harmful Pb(II) ions from polluted water (Vakili et al., 2019; Wang et al., 2019; Hoang et al., 2020). Among them, adsorption is commonly recognized as a potential method to remove various pollutants (Au, 2020; Dong et al., 2020; Tamas et al., 2020). Using adsorption materials to remove heavy metal pollutants in water through physical or chemical action is considered to be one of the effective ways to eliminate Pb(II) pollution (Ezeokonkwo et al., 2018; Hashem et al., 2020). The design and development of new adsorbents with high adsorption capacity and fast adsorption rate have received considerable retention in both academic and industrial domains.

Hydrogel is a special polymer material with a moderately cross-linked network structure, which contains abundant functional groups in its unique network (Gull et al., 2020). The functional groups in the hydrogel’s three-dimensional network will adsorb metal ions into the network through hydrogen bonds, electrostatic force and chemical complex interaction, and the network space will accommodate these adsorbed ions, so as to achieve high-capacity adsorption. Therefore, hydrogel adsorbents with higher adsorption capacity, faster adsorption rate and excellent desorption-regeneration ability are commonly regarded as highly efficient adsorbents. With the increasing attention on the environmental friendliness of materials, people are more inclined to use natural polymers to synthesize hydrogel materials for various purposes, because natural polymers have the advantages of being renewable, cheap, non-toxic and environmentally friendly (Wang et al., 2017; Kaur et al., 2020).

Sodium alginate (SA) is a water-soluble anionic biopolymer extracted from natural seaweed or produced by bacteria. Its structure is composed of α-1, 4-L-glucuronic acid (G unit) and poly-β-1, 4-D-mannuronic acid (M unit) with different ratios of 1–4. Because SA has the advantages of being renewable, abundant, safe, non-toxic, and biodegradable, it can be modified by ionic complex cross-linking, graft polymerization and chemical cross-linking reactions, so as to obtain new products with better performance (Yadav et al., 2011; Yadav and Rhee, 2012). In addition to the environmental friendliness of SA, the carboxyl groups on its molecular chain can serve as an active site for adsorbing metal ions, and the reactive -OH group on its molecular chain can also be easily modified by grafting reaction to obtain hydrogel materials with more functional groups (Wang and Wang, 2010). Due to the superior performance and eco-friendly advantages of SA, it has been widely used to prepare various polymer packing films (Saravanakumar et al., 2020; Zhang et al., 2020), drug-delivery vehicles (Mishra et al., 2021), antibacterial materials (Dwivedi et al., 2020), carrier of catalyst (Khan et al., 2020), superabsorbent hydrogel (Kassem et al., 2020), coatings (Fang et al., 2021) and adsorbent (Wang et al., 2013a; Gao et al., 2020). These materials derived from SA not only show excellent performance, but also exhibit unique eco-friendly advantages. Therefore, the SA-based polymer materials have received increasing attention for the decontamination of various pollutants from polluted water. The current focus is on improving the gel strength, regeneration capability and adsorption performance by introducing functional groups *via* grafting polymerization reaction or other functional components.

Semi-interpenetrating polymer network (semi-IPN) hydrogel is a polymer material with a structure similar to an “alloy”, which is composed of a chemically cross-linked polymer network and a linear polymer chain penetrating the polymer network. Due to the simultaneous presence of chemical crosslinking and physical crosslinking in this type of hydrogel, it has mechanical strength better than traditional hydrogels (Myung et al., 2008; Wang et al., 2013; Zhang et al., 2015). Polyvinyl alcohol (PVA) is a widely used non-toxic, biodegradable, water-soluble linear polymer, which has the inherent advantages of preparing ideal “green” materials (Yang et al., 2011). The polymer material prepared with PVA is not only non-toxic, but also has good biocompatibility, good viscoelasticity, strength and processability (Sahoo et al., 2002; Huang et al., 2012). PVA hydrogels have been used potentially in biomedicine and packaging, such as wound dressings (Kim, 2008), artificial articular cartilages (Kobayashi and Oka, 2004), drug delivery (Ossipov et al., 2013), and antibacterial material (Rafiq et al., 2018). PVA hydrogels can be prepared through physical or chemical crosslinking approach, and can also blend with other polymer chains to form composite gels. The neat chemically crosslinked hydrogel usually shows poor flexibility because the polymer chains were locked by chemical crosslinking points, which can’t effectively transfer stress when the hydrogel being stretched (Liu et al., 2015). As a result, the hydrogel is brittle at swelling state, and is easily to be broken. It has been proven that PVA can form physical crosslinking with other polymer chains by non-covalent bonding interaction, which can effectively transfer stress when it was stretched or compressed by external forces. Therefore, incorporating PVA polymer chains into the cross-linked polymer network can improve the regularity of the network and increase the strength, hydrophilicity and other functional properties of the hydrogel network (Dragan, 2014). In light of the excellent advantages of SA and PVA, the semi-IPN hydrogel with improved gel strength and adsorption performance will be formed by combination of SA-*g*-PNaA and PVA *via* grafting polymerization reaction in water medium.

Based on the background mentioned above, the sodium alginate-*g*-poly(sodium acrylate)/poly(vinyl alcohol) (SA-*g*-PNaA/PVA) semi-IPN hydrogels was designed and synthesized in aqueous solution by free-radical grafting polymerization and crosslinking reactions. The as-prepared hydrogels were characterized by thermogravimetric analysis (TGA), Fourier transform infrared spectra (FTIR), and scanning electronic microscope (SEM). In addition, the role of PVA on improving the adsorption capacity for Pb(II), the gel strength and regeneration ability of the hydrogel was studied. The adsorption isotherm of the hydrogels towards Pb(II) were evaluated systematically. The adsorption kinetics and adsorption mechanism was explored.

## Experimental

### Materials

Sodium alginate (SA) (kinetic viscosity of 1.0 wt% aqueous solution, 20 cp), *N, N’*-methylene-*bis*-acrylamide (MBA, AR grade) and PbCl_2_ (AR grade) was purchased from Sinopharm Chemical Reagent Co., Ltd., Shanghai, China. Acrylic acid (AA) and ammonium persulfate (APS, AR grade) were supplied by Shanghai Regent Corp., Shanghai, China. PVA (Molecular weight, 85,000–124,000; alcoholysis degree is 95.5–96.5%) was purchased from Acros and used as received. All the other reagents used in this experiment are of analytical grade. All the chemicals used for this study were used as received. All the aqueous solutions are prepared with deionized water.

### Synthesis of SA-*g*-PNaA/PVA Hydrogels

At a temperature of 95°C, 30 ml of water and a predetermined amount of PVA (accounting for 2, 5, 10, 15, and 20% of the total mass of monomer AA and SA) were added in a four-necked flask equipped with a mechanical stirrer, a nitrogen tube, and a reflux condenser, and the mixture was stirred well to dissolve PVA to obtain a transparent solution. After the solution was cooled to 60°C, 1.0 g of SA powder was added thereto, and the mixture was continuously stirred until a uniform aqueous solution was formed. Then, under the protection of nitrogen, 10 ml of the aqueous solution containing 0.12 g of initiator APS was added, and the reactants were kept at 60°C for 10 min to decompose the initiator to generate free radicals. Immediately afterwards, a mixed solution containing 7.2 g acrylic acid (AA), 8 ml 8.0 mol/L NaOH solution and 72 mg cross-linking agent MBA was added to the flask under stirring, and the temperature was slowly raised to 70°C and kept at this temperature for 3 h to complete the polymerization reaction. After freezing the obtained hydrogel sample at −20°C, it was immersed in absolute ethanol for dehydration, and finally a dried product was obtained. The dried hydrogel was crushed and passed through a 100 mesh sieve for further use. According to a similar method, without adding PVA, the control sample SA-*g*-PNaA hydrogel was prepared. According to the amount of PVA, the SA-*g*-PNaA/PVA hydrogels are labeled as Semi-IPN2, Semi-IPN5, Semi-IPN10 and Semi-IPN20, respectively.

### Measurement of Gel Strength

The gel strength of hydrogel was tested by a previously reported rheological method, specifically the storage modulus and loss modulus of the hydrogel in the swollen state (water content controlled at 98%) was determined using a Physica MCR 301 rheometer (Germany) at 25° (Ramazani-Harandi et al., 2006). A parallel plate with the diameter of 25 mm was used to determine storage modulus (G’). The deformation strain was fixed at 0.5%, and the angular frequency (*ω*, rad/s) was fixed at 10 rad/s. The averages of three test results were reported in this paper.

### Evaluation of Adsorption Performance

The following procedures were employed to test the adsorption performance of the hydrogel adsorbent. Typically, 50 mg of the adsorbent was mixed with 25 ml of the aqueous solutions of Pb(II). Then, the mixture was shaken in a constant temperature shaker (THZ-98A, Yiheng, Shanghai, China) at a speed of 120 rpm and at 30°C for 120 min to make the adsorbent reach adsorption equilibrium. The solid adsorbent was rapidly separated from the aqueous Pb(II) solution by using a 0.22 μm film filter. The concentration of Pb(II) ions in the filtered solution was determined with an atomic absorption spectrometry (Z-8000 AAS, Hitachi, Japan). The adsorption amount of Pb(II) onto unit mass of hydrogel adsorbent was calculated by the Eq. 1.

$$q=\left(C_{o}-C_{e}\right)V/m$$ (1)

Here, *q* represents the adsorption amount of Pb(II) on the adsorbent at time *t* (marked as *q*_t_) or at equilibrium state (marked as *q*_e_), and its unit is “mg/g”; *C*_0_ and *C*_e_ represent the concentration of Pb(II) ions before and after adsorption, and its unit is “mg/L”; *V* represents the volume of Pb(II) solution after adsorption, and its unit is “L”; *m* represents the mass of the hydrogel adsorbent used for adsorption, and its unit is “g”.

In this work, the adsorption isotherm of the adsorbent for Pb(II) was tested in the initial concentration range of 880–3,200 mg/L (pH 5). Under the condition of the initial Pb(II) solution concentration of 1,150 mg/L, the adsorption performance of the adsorbent under different pH values (2.0–5.0) was tested, and the effect of pH on the adsorption behavior was studied. The pH value of the solution is adjusted with 0.1 mol/L HCl aqueous solution or NaOH solution. Use Eq. 1 to calculate the adsorption capacity of the hydrogel adsorbent. All adsorption experiments were performed 6 times in parallel, and the average of the data obtained was used for analysis and discussion. In order to investigate the adsorption isotherm of the hydrogel adsorbent and reveal how does the adsorbent interact with Pb(II) ions, the adsorption experiment data was fitted with Langmuir (Eq. 2) (Langmuir, 1916) and Freundlich (Eq. 3) (Freundlich, 1906) adsorption isotherm models.

$$C_{e}/q_{e}=1/q_{m}b+C_{e}/q_{m}$$ (2)

$$\log q_{e}=\log K+\left(\mathrm{1/}n\right)\log C_{e}$$ (3)

In these equations, *q*_e_ denotes the adsorption capacities of the adsorbents towards Pb(II) at equilibrium state, and its unit is “mg/g”; *C*_e_ denotes the residual concentration of Pb(II) solutions after being adsorbed with the hydrogel adsorbent, and its unit is “mg/L”. *q*_m_ refers to the saturated adsorption capacity of the hydrogel adsorbent, and its unit is “mg/g”. *b* is the Langmuir adsorption constant (L/mg) related to the free energy of adsorption. *K* (L/g) and n (dimensionless) represent Freundlich isotherm constant and heterogeneity factor, respectively.

The kinetic adsorption properties were tested according to the following procedure. Mixing 25 ml of Pb(II) solution (concentration is 1,150 mg/L) and 50 mg of adsorbent thoroughly, and then the mixture was filtered through a 0.22 μm membrane filter at different time intervals (10–3,600 s) to make the solid adsorbent and the solution separate. The concentration of residual Pb(II) in the filtrate was determined by atomic absorption spectrometry and the adsorption capacities of the hydrogel adsorbent at each time interval were calculated according to Eq. 1. In order to study the adsorption kinetic of the adsorbent, the adsorption experimental data were fitted with pseudo-first-order model (Eq. 4) (Ho, 2004) and pseudo-second-order model (Eq. 5) (Ho and McKay, 1998), respectively.

$$\log\left(q_{e}-q_{t}\right)=\log\;q_{e}-\left(k_{1}/2.303\right)t$$ (4)

$$t/q_{t}=1/K_{2}\times qe^{2}+t/qe$$ (5)

In the two equations, *q*_e_ and *q*_t_ denote the adsorption amount of Pb(II) on unit mass of adsorbent at the equilibrium adsorption state and the adsorption time *t*, respectively, and their units are “mg/g.” *k*_1_ and *k*_2_ respectively refer to the rate constants obtained by fitting the pseudo-first-order kinetic model and the pseudo-second-order kinetic model.

### Measurement of Desorption and Regeneration Properties

Disperse 50 mg of adsorbents (SA-*g*-PNaA, Semi-IPN2 and Semi-IPN10, respectively) into 25 ml of Pb(II) solution (initial concentration is 1,150 mg/L, pH is 5) to reach equilibrium adsorption state. The adsorbent after adsorbing Pb(II) is filtered out, and re-dispersed in 25 ml of HNO_3_ aqueous solution (0.1 mol/L) under magnetic stirring. After that, the adsorbent was separated from the aqueous solution by centrifugation. The obtained adsorbent was regenerated with 0.5 mol/L NaOH aqueous solution, followed by washing with deionized water 3 times, and then dried in an oven at 70°C and used for the next adsorption cycle. Atomic absorption spectrometry was used to determine the content of Pb(II) in the desorption solution to calculate the recovery rate. The adsorption and desorption treatment was carried out 5 times in succession, and the adsorption capacity of the adsorbent and recovery rate of Pb(II) with different regeneration times were obtained.

### Characterizations

A Thermo Nicolet NEXUS TM spectrophotometer was used to collect the FTIR spectra in the wavenumber region of 4,000–400 cm^−1^. The sample for test was uniformly mixed with KBr by a gentle grinding process, and then pressed into disc-shaped KBr platelet. A field emission scanning electron microscope (FE-SEM, JSM–6701F, JEOL, Ltd., Japan) was employed to observe the morphologies of samples. Before observation, the sample was coated with gold film to enhance the conductivity of sample. The concentration of Pb(II) in the lixivium of the composites was determined by a Z-8000 AAS atomic absorption spectrophotometer (Hitachi, Japan). Thermogravimetric analysis (TGA) was performed on the Diamond TG-DTA 6300 thermal analyzer, the test temperature is 30∼800°C, the gas atmosphere is *N*_2_, and the heating rate is 10°C/min.

## Results and Discussion

### FTIR Spectra Analysis

**FIGURE 1:**
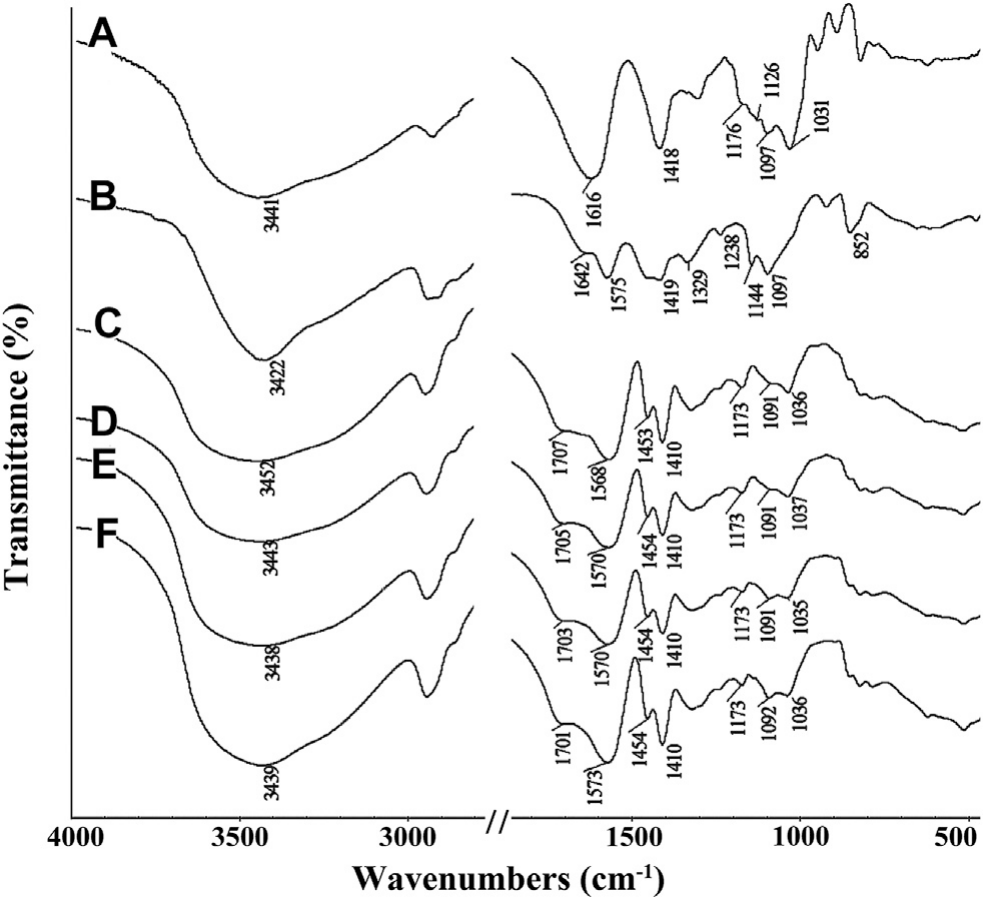
FTIR spectra of **(A)** SA, **(B)** PVA, **(C)** Semi-IPN2, **(D)** Semi-IPN5, **(E)** Semi-IPN10, **(F)** Semi-IPN20.

The FTIR spectra of SA, PVA, SA-*g*-PNaA and the SA-g-PNaA/PVA hydrogels with different amounts of PVA are shown in Figure 1. The absorption peaks of SA at 1,616 cm^−1^, 1,418 cm^−1^, 1,176 cm^−1^, and 1,097 cm^−1^ were ascribed to the O-C=O asymmetric stretching vibration, the O-C=O symmetric stretching vibration, the stretching vibration of C-O-C and the C-OH stretching vibration, respectively (Figure 1A) (Khalid et al., 2018). After the polymerization reaction, the new absorption peak at 1,707 cm^−1^ (the C=O stretching vibration of–COOH groups) were observed in the FTIR spectra of the SA-*g*-PNaA/PVA hydrogel adsorbent (Figures 1C–F), and the C-OH stretching vibration peak of SA at 1,031 cm^−1^ significantly weakened and shifted to 1,035–1,037 cm^−1^, indicating the C-OH groups of SA participate in grafting polymerization reaction, and the PNaA chains were grafted onto the SA molecular chains (Wang et al., 2013a). The characteristic absorption peaks of PVA were observed at 1,642 cm^−1^ (C=O stretching of ester groups), 1,419 cm^−1^ (-OH deformation), 1,144 cm^−1^ (C-O-C stretching vibration), and 1,097 cm^−1^ (C-OH stretching vibration) (Mansur et al., 2004). After polymerization reaction, the absorption peaks of PVA at 1,144 cm^−1^ and 1,097 cm^−1^ shift to 1,091 cm^−1^ and 1,036 cm^−1^ (also ascribed to the overlapping of the peak of PVA at 1,097 cm^−1^ and the peak of SA at 1,031 cm^−1^), respectively. In addition, the C=O stretching vibration peak of PVA at 1,575 cm^−1^ shifted to 1,568–1,573 cm^−1^ after grafting polymerization (Figures 1B–F). The above results indicate that hydrogen bond interaction occurs between PVA chains and the SA-*g*-PNaA polymer network (Dong et al., 2006; Sun and Uyama, 2013).

### Morphological Analysis

**FIGURE 2:**
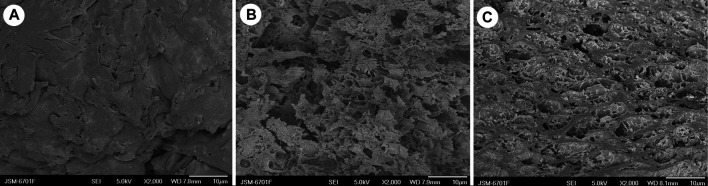
SEM micrographs of **(A)** SA-*g*-PNaA, **(B)** Semi-IPN2, and **(C)** Semi-IPN10.

As is shown in Figure 2, the SA-*g*-PNaA hydrogel shows a dense surface with little wrinkles, pores and gaps (Figure 2A). However, loose surface and numerous pores were observed in the SEM micrographs of the Semi-IPN2 and Semi-IPN10 hydrogels (Figures 2B,C), indicating the incorporation of PVA is favorable to the formation of porous structure. For chemically crosslinked SA-*g*-PNaA hydrogel, the polymer chains were fixed by covalent crosslinking point, so the flexability of gel network is poor, which may strongly shrink when it was dried to form a relatively dense surface. After incorporation of PVA, the flexability of gel was improved, so the drying process does not induce the great change of network voids. After water was removed, the three-dimensional network still remains well, and thus the macroporous hydrogel was obtained.

### DTG and DSC Analysis

**FIGURE 3:**
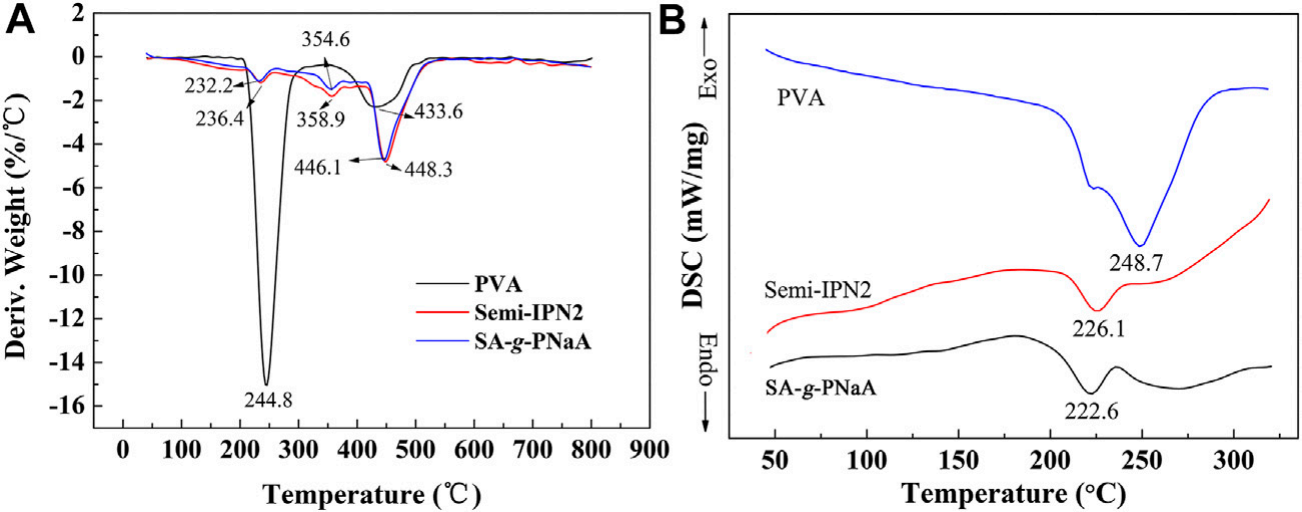
DTG curves **(A)** and DSC curves **(B)** of PVA, SA-*g*-PNaA and Semi-IPN2 adsorbent.

The DTG diagrams of PVA, Semi-IPN2 and SA-*g*-PNaA are shown in Figure 3A. It can be seen from the DTG curve that the T_d_ (decomposition temperature corresponding to the maximum mass loss rate) values of PVA, Semi-IPN2 and SA-*g*-PNaA appear at 244.8°C, 446.1°C, and 448.3°C, respectively. This indicates that the incorporation of PVA into the SA-*g*-PNaA network facilitates to improve its thermal stability because the -OH groups on PVA may form strong hydrogen bonding interaction or physical crosslinking points with the polymer chains in SA-g-PNaA network, which may limit the movement of linear polymer chain when it was heated. With the increase of PVA content, the T_d_ value also increased, due to the intensified interaction between PVA and the polymer matrix. After incorporation of 2 wt% PVA, the thermal decomposition temperature (centered at 232.2°C) of SA-*g*-PNaA at the first stage on DTG curves increased to 236.4°C, and the thermal decomposition temperature (centered at 354.6°C) of SA-*g*-PNaA at the second stage on DTG curves increased to 358.9°C, indicating that the incorporation of PVA improve the thermal stability of the Semi-IPN hydrogel by intensifying the interaction among polymer chains.

As seen from Figure 3B, an endothermic peak at 248.7°C (ascribed to the dissociation process of hydrogen bonds among chains) was observed on the DSC curve of PVA. The endothermic peak of SA-*g*-PNaA appears at 222.6°C. When the PVA was incorporated into the crosslinked SA-*g*-NaA network to form semi-IPN SA-*g*-PNaA/PVA hydrogel, the endothermic peak increased to 226.1°C (for Semi-IPN2). The increase of endothermic peak means the dissociation process of hydrogen bonds becomes difficult, and implied that the stronger tangle action between PVA chains and SA-*g*-PNaA network (Kulkarni et al., 2010).

### Effect of PVA Content on the Adsorption Capability and Gel Strength

**FIGURE 4:**
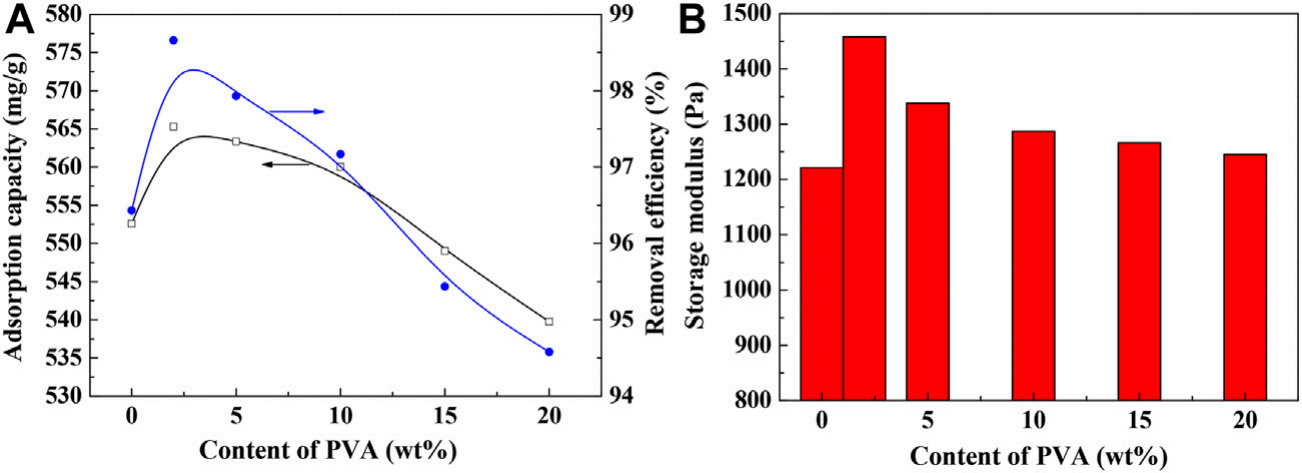
Effect of PVA content on the adsorption capacity **(A)** and the gel strength **(B)**. Adsorption conditions: initial Pb(II) concentration, 1,150 mg/L; adsorbent dosage, 2 g/L; adsorption time, 120 min; pH, 5.0; adsorption temperature, 30°C.

The introduction of PVA containing large amounts of hydroxyl groups into the SA-*g*-PNaA hydrogel network may affect the network formulation of the hydrogel, and thus affect its adsorption capacity and gel strength. As shown in Figure 4A, the adsorption capacity and removal efficiency of the adsorbents towards Pb(II) initially increased with increasing the content of PVA to 2 wt%, and then decreased with the further increase of PVA content. The adsorption capacities of SA-*g*-PNaA and Semi-IPN2 toward Pb(II) are 554.53 mg/g and 568.99 mg/g, respectively, and the corresponding removal ratio of Pb(II) is 97.12 and 98.39% (initial Pb(II) concentration, 1,150 mg/L; pH 5), respectively.

As shown in Figure 4B, the incorporation of PVA has also contributed to improve the gel strength of the hydrogel adsorbents at wet state. The gel strength of the hydrogel increased by 19.4% after incorporation of 2 wt% of PVA. The increase of gel strength can be ascribed to the enhancement of the interaction among PVA chains and the grafting polymer chains (Li et al., 2018). PVA chains containing numerous –OH groups and –C=O groups was interpenetrated within the polymer network, which may form hydrogen bonding interaction with the –COO^–^, -OH, and -COOH groups in the SA-*g*-PNaA network and tangled with the grafted polymer chains to generate a physical crosslinking domain. The additional physical crosslinking is favorable to improve the viscoelasticity of the hydrogel, and thus enhance the gel strength of hydrogel (Pita-López et al., 2021). The increase of gel strength may improve the stability of network structure, and enhance the adsorption capacity and reusability of the hydrogel adsorbent.

### Analysis of Adsorption Isotherms

**FIGURE 5:**
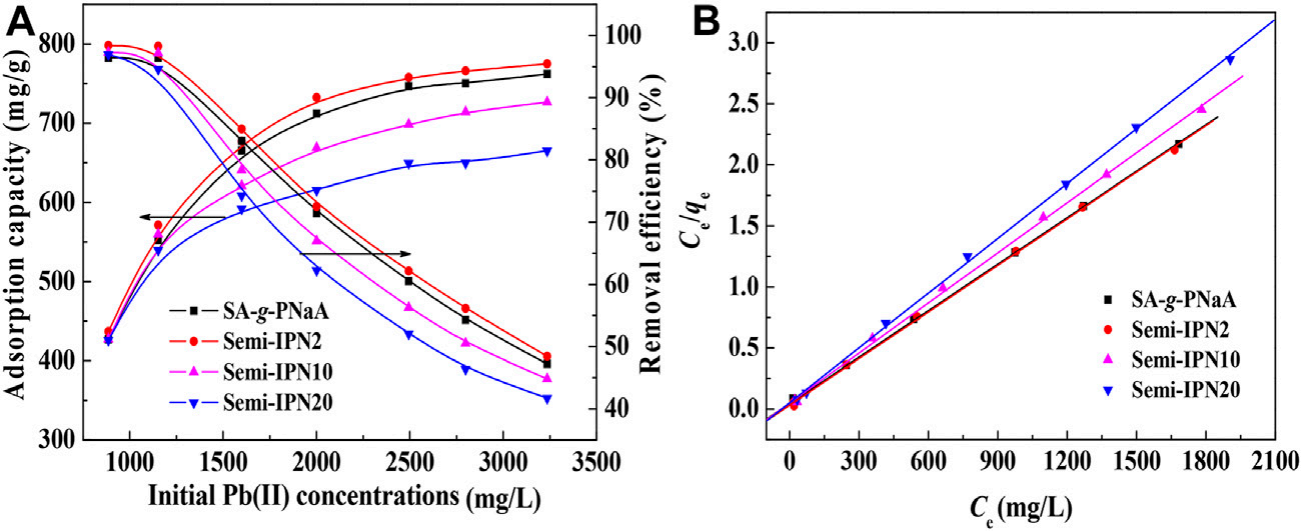
**(A)** Variations of adsorption capacity and removal efficiency of the adsorbent for Pb(II) as a function of initial concentration; **(B)** Plots of *C*_e_/*q*_e_ vs. *C*_e_ obtained by fitting with Langmuir isotherm model. Adsorption conditions: pH, 5.0; adsorbent dosage, 2 g/L; adsorption time, 120 min; adsorption temperature, 30°C.

Figure 5 shows the dependence of adsorption capacity on the initial Pb(II) concentration and the curves fitting with Langmuir model. It is obvious that the adsorption capacity increased with increase of initial Pb(II) concentration, until equilibrium adsorption was attained at the initial concentrations of 3,200 mg/L. This is because as the concentration of Pb(II) ion in the external solution increases, a larger concentration gradient is generated between the bulk solution and the adsorbent, which makes the Pb(II) in the solution diffuse to the adsorbent and combine with the adsorbent more easily, so the adsorption capacity also increases (Xiong et al., 2010). On the contrary, as the initial Pb(II) concentration increases, the total amount of Pb(II) in the solution will also increase, and the removal rate of Pb(II) from the solution decreases. When the amount of Pb(II) adsorbed by the adsorbent is less than the amount of Pb(II) increased in the solution, the removal ratio will decrease. It is essential to interpret the adsorption behavior and predict the extent of adsorption by fitting the equilibrium adsorption data with Langmuir (Eq. 2) model, and Freundlich model (Eq. 3). As shown in Figure 5B, the plots of *C*_e_/*q*_e_ vs. *C*_e_ exhibits perfect straight plots with *R* ^2^ > 0.9997. The parameters listed in Table 1 can be obtained by fitting adsorption data to the Langmuir (plotting *C*_e_/*q*_e_ vs. *C*_e_) and Freundlich (plotting log*q*_e_ vs. log*C*_e_) adsorption isotherm models. It was noticed from Table 1 that the adsorption capacity calculated by fitting with Freundlich isotherm model (*R* ^2^ < 0.9447) displays obvious difference with the experimental value; whereas the adsorption capacity obtained by fitting with Langmuir adsorption isotherm model (*R* ^2^ > 0.9997) is close to the experimental value. This indicates that the adsorption process of the adsorbent for Pb(II) is in good agreement with the Langmuir isotherm model, and that Pb(II) is adsorbed onto the adsorbent through a single layer covering. The saturation adsorption capacities of Semi-IPN2 and Semi-IPN10 are determined to be 784.97 mg/g and 726.66 mg/g, respectively, which are better than most of other adsorbents (Table 2).

**TABLE 1:** Adsorption isotherm parameters for the adsorption of Pb(II) ions onto the hydrogel adsorbent.

Samples	*q*_e_ (mg/g)	Langmuir equation			Freundlich equation		
		*q*_m_ (mg/g)	*b* (L/mg)	*R* ^2^	*K*	*n*	*R* ^2^
SA-*g*-PNaA	775.92	781.25	0.0460	0.9998	368.03	9.558	0.9479
Semi-IPN2	784.97	787.40	0.0434	0.9997	372.88	9.708	0.9414
Semi-IPN10	726.66	729.93	0.0307	0.9996	351.61	10.189	0.9338
Semi-IPN20	665.05	666.67	0.0299	0.9997	328.22	10.487	0.9513

**TABLE 2:** Comparison of the adsorption capacities of different adsorbents for Pb(II).

Adsorbents	*Adsorption conditions*	*q*_*e*_ (mg/g)	Ref.
Polyacrylic acid grafted magnetic chitosan	Initial concentration, 500 mg/L; adsorbent dosage, 0.5 g/L; pH 5.48	204.89	Hu et al. (2020)
Spherical mesoporous silica	Initial concentration, 120 mg/L; adsorbent dosage, 1 g/L; pH 4	59.03	Zhu et al. (2016)
Hierarchical MgFe_2_O_4_ microspheres	Initial concentration, 250 mg/L; adsorbent dosage, 0.5 g/L; pH 7	113.70	Kang et al. (2015)
Halloysite/alginate nanocomposite beads	Initial concentration, 200 mg/L; pH 5	325.00	Chiew et al. (2016)
Hydroxyapatite/chitosan porous materials	Initial concentration, 400 mg/L; pH 5.5	264.42	Lei et al. (2015)
CS coated cotton fiber	Initial concentration, 2,072 mg/L; pH 6.5	101.53	Zhang et al. (2008)
Amine functionalized rice husk magnetic nanoparticle biocomposites	Initial concentration, 350 mg/L; pH 5	680.19	Nata et al. (2020)
Alginic acid (AmAA)	Initial concentration, 800 mg/L; pH = 5, adsorbent dose = 0.25 g/L; time = 30 min	535.87	Vaid et al. (2020)
Semi-IPN2	Initial concentration, 1,150 mg/L; adsorbent dosage, 2 g/L; pH 5	568.99	This work
Semi-IPN2	Initial concentration, 3,200 mg/L; adsorbent dosage, 2 g/L; pH 5	784.97	This work

### Adsorption Kinetics

**FIGURE 6:**
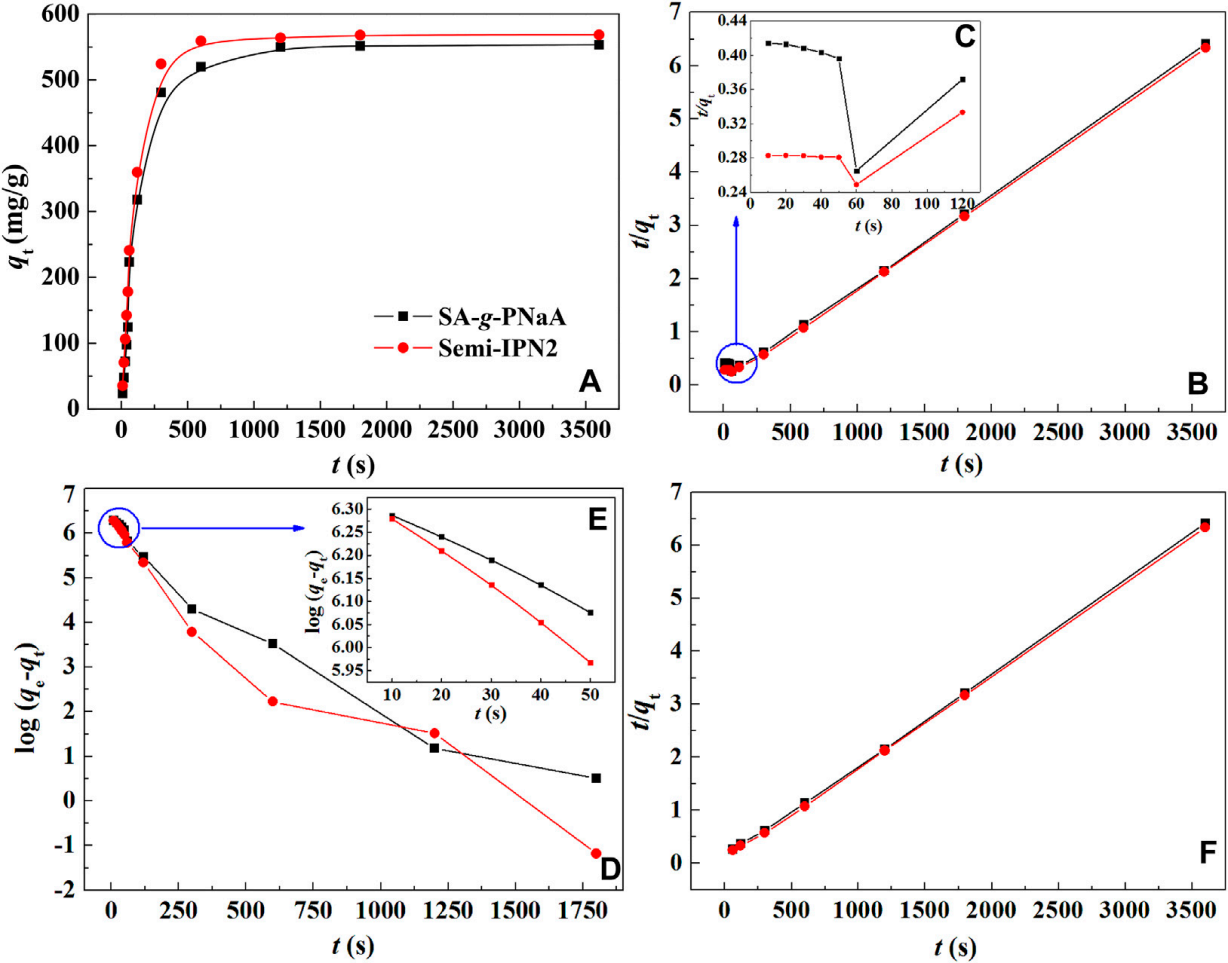
**(A)** Adsorption kinetic curves of SA-*g*-PNaA and Semi-IPN2 hydrogels; **(B)** fitting curves of *t*/*q*_t_ *versus t*; **(C)** the fitting curves of *t*/*q*_t_ *versus* t (≤120 s); **(D)** curves of log(*q*_e−_ *q*_t_) *versus t*; **(E)** curves of log (*q*_e−_ *q*_t_) *versus* t (≤50 s); **(F)** fitting curves of *t*/*q*_t_ *versus* t (>120 s).

An ideal adsorption rate is the basic prerequisite to ensure the practical performance of the adsorbent. The type and number of functional groups, pore structure and polymer network structure are the main factors affecting the adsorption rate of hydrogel materials. As shown in Figure 6A, in the initial stage, the adsorption capacity of Pb(II) by the adsorbent increases rapidly, and then the adsorption rate gradually slowed until the equilibrium adsorption was reached within 10 min. Comparatively, the semi-IPN hydrogel adsorbent has relatively rapid adsorption rate than the SA-*g*-PNaA hydrogel due to its relatively better porous structure. In order to study the adsorption kinetics of the adsorbent, the adsorption kinetic models of the pseudo-second-order (Eq. 4) and pseudo-first-order (Eq. 5) were used to fit the experimentally measured adsorption data. Figure 6B gives the fitting curves of the hydrogel adsorbents using pseudo-second-order kinetic model. It was noticed that the fitting result at the shorter time (*t* ≤ 50 s) is not a perfect straight line (Figure 6C), but the fitting curve at the time larger than 120 s exhibits a perfect line (*R* ^2^ = 1.0000) (Figure 6F). This indicates that the adsorption process of the hydrogel at the later part strictly follows the pseudo-second-order kinetic model very well. However, the linear correlation of the curve obtained by fitting the adsorption data at this stage with pseudo-first-order kinetic model is very poor (Figure 6D). Interestingly, the fitting curve of log(*q*_e_−*q*_t_) *versus t* (≤50 s) with pseudo-first-order kinetic model give the perfect straight line with the *R* ^2^ values of 0.9988 and 0.9990 for SA-*g*-PNaA and Semi-IPN2, respectively (Figure 6E; Table 3), but a poor line correlation with the *R* ^2^ value <0.9721 was obtained for the whole adsorption process (Figure 6D; Table 3). It can be noticed that the *q*_e_, _cal_ value calculated with pseudo-second-order model (*t* ≥ 60 s) is close to that calculated with the pseudo-first-order model (*t* ≤ 50 s), and these values are more close to the experimental values. The results show that when the adsorption time *t* ≤ 50 s, the kinetic behavior of the adsorbent to adsorb Pb(II) obeys both the pseudo-first-order kinetic model and the pseudo-second-order kinetic model. During the initial adsorption process, the physical diffusion adsorption and chemical adsorption contribute to Pb(II) adsorption. As a whole, the physical diffusion adsorption is dominant at initial stage (*t* ≤ 50 s) and chemical adsorption is dominant at the following stage (*t* ≥ 60 s), and the inflection point of these two processes appears at 60 s.

**TABLE 3:** Adsorption kinetic parameters for adsorption of Pb(II) on the hydrogel.

Samples	Pseudo-first-order model				Pseudo-second-order model			
	*q*_*e*_, _exp_	*q*_m_, _cal_	*K*_1_	*R* ^2^	*q*_m_, _cal_	*k*_2_ × 10^5^	*k*_2i_	*R* ^2^
	mg/g	mg/g	s^−1^		mg/g	g/(mg∙s)	mg/(g∙s)	
SA-*g*-PNaA	553.57	418.14	0.0080	0.9721	595.24	1.116	3.953	0.9981
Semi-IPN2	568.99	383.82	0.0095	0.9685	591.72	1.699	5.947	0.9991
*t* ≤ 50 s, for Pseudo-first-order model					*t* ≥ *60* *s,* for Pseudo-second-order model			
SA-*g*-PNaA	553.57	568.75	0.0121	0.9988	574.71	2.609	8.616	0.9998
Semi-IPN2	568.99	580.04	0.0179	0.9990	579.04	2.582	8.627	1.0000

As shown in Table 3, the adsorption rate constant of the SA-*g*-PNaA hydrogel is 0.0121 s^−1^ at initial stage, but increased to 0.0179 s^−1^ for Semi-IPN2. The pseudo-second-order kinetic constant of SA-*g*-PNaA is 3.953 mg/g·s, but increased to 5.947 mg/g·s for Semi-IPN2. These results indicate that the introduction of PVA may clearly increase the adsorption rate of the hydrogel, which is favorable to the practical application of hydrogel. The main reason may be that the PVA improved the network structure of the hydrogel, and make the network becomes regular. In addition, the incorporation of PVA can improve the porous morphology of the as-prepared hydrogel, which can reduce the transport resistance of metal ions in the polymer network, thereby increasing the adsorption rate.

### pH-Responsive Adsorption Behavior and Mechanisms

**FIGURE 7:**
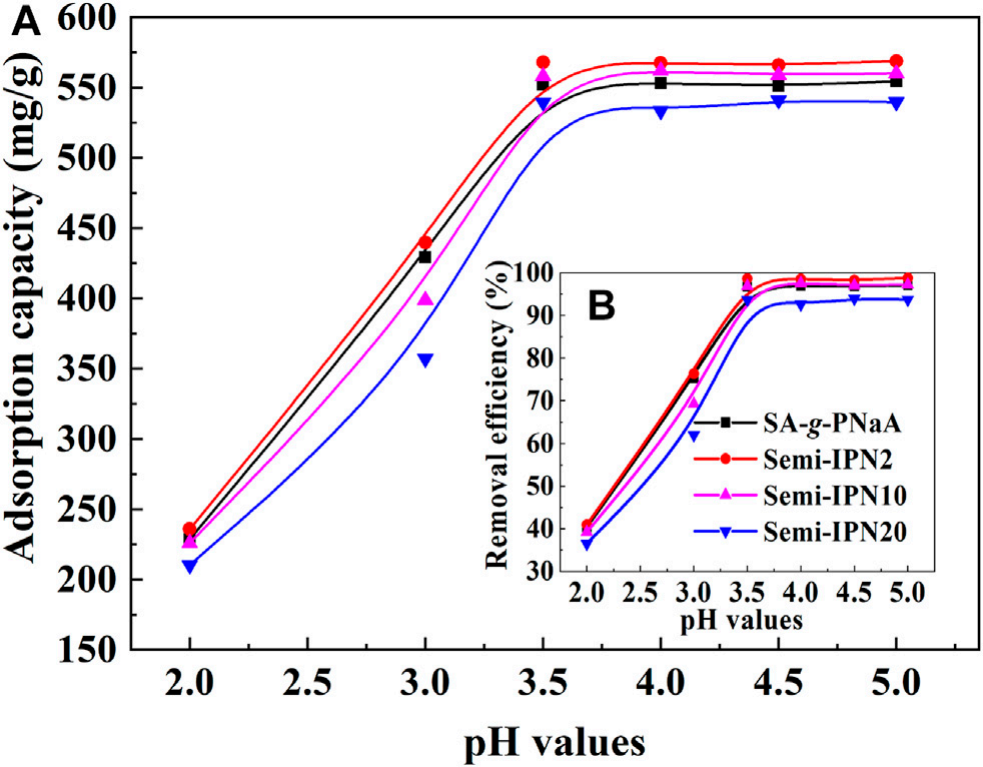
Variable curves of adsorption capacity **(A)** and removal efficiency **(B)** against the change of pH values.

The main reason why the hydrogel adsorbent can efficiently adsorb Pb(II) ions is the complexation of the functional groups in the network structure to Pb(II) and the electrostatic attraction of the network structure to Pb(II) ions. The change of external pH values may affect the existence stage of functional groups and thus affect the adsorption behaviors. As is shown in Figure 7, the adsorption capacity and removal efficiency of the hydrogel adsorbents for Pb(II) sharply increased with increasing the external pH values, and almost keep constant at pH above 3.5. The maximum adsorption capacity of the Semi-IPN2 adsorbent sample at pH 5 is 568.99 mg/g (Figure 7), and the corresponding removal rate is 98.39%. The main reasons can be ascribed to the transformation between–COOH and -COO^–^ groups. The pKa value of poly(acrylic acid) is about 4.7 (Chen et al., 2008), so the–COO^–^ groups on the hydrogel adsorbent were transformed as–COOH groups at acidic pH (<3.5). The–COOH groups may form strong hydrogen bonds with the functional groups in the hydrogel network to form additional crosslinking, and thus the network shrinks and its capture capability to metal ions decreased. Also, the–COOH groups have relatively weaker complexing ability to Pb(II) ions than -COO^-^ groups, so the increase of -COO^-^ amounts causes the increase of adsorption capacity (Astrini et al., 2015). In addition, the increase of the negative charge on the surface of the adsorbent with increasing pH is also one of the reasons for the enhancement of adsorption capacity. At pH 2, the Zeta potential of the adsorbent is +2.6 mV. When the pH value increases to 3.5 and 5, the Zeta potential is −9.6 mV, and −20.21 mV, respectively. As the pH of the solution increases, the Zeta potential of the adsorbent becomes more negative. This is conducive to the adsorbent’s ability to efficiently adsorb positively charged metal ions.

**FIGURE 8:**
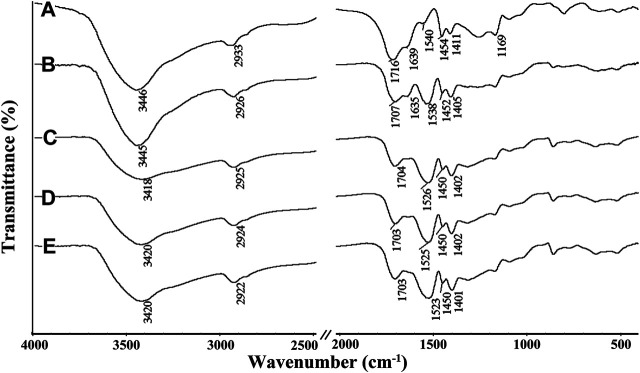
FTIR spectra of Semi-IPN2 adsorbent after adsorption of Pb(II) at the pH values of **(A)** 3, **(B)** 3.5, **(C)** 4, **(D)** 4.5, and **(E)** 5.

**SCHEME 1:**
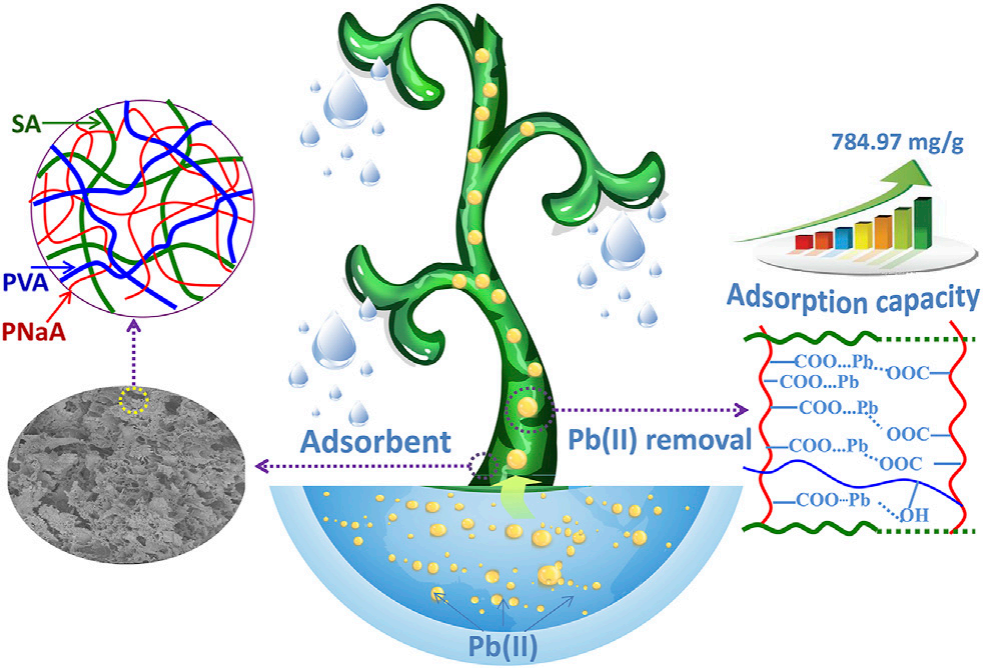
Scheme illustrated the gel network structure of the adsorbent and its adsorption actions for Pb(II).

In order to explore the adsorption mechanism, the FTIR spectra of the Semi-IPN2 hydrogel adsorbent after Pb(II) adsorption under different pH conditions were analyzed. As shown in Figure 8, after adsorbing Pb(II) at different pH values, the characteristic absorption peak of the adsorbent changed significantly. The characteristic bands of the adsorbent appear at 1707 cm^−1^ (C = *o* stretching vibration of -COOH groups), 1,568 cm^−1^ (asymmetric stretching vibration of -COO^-^ groups), 1,453 cm^−1^ and 1,410 cm^−1^ (symmetrical stretching vibration of -COO^-^ groups), 1,091 cm^−1^ (C-O-C stretching vibration), 1,036 cm^−1^ (C-OH stretching vibration). After adsorption of Pb(II) at pH 3, 3.5, 4, 4.5, and 5, the C=O absorption bands of -COOH groups in the adsorbent shift from 1,707 cm^−1^ to 1,716 and 1,639 cm^−1^, 1,707 cm^−1^, 1,704 cm^−1^, 1,703 cm^−1^, and 1,703 cm^−1^, respectively; the bands of–COO^-^ groups at 1,568 cm^−1^ shift to 1,540 cm^−1^, 1,538 cm^−1^, 1,526 cm^−1^, 1,525 cm^−1^, and 1,523 cm^−1^, respectively. The shift of the C = o stretching vibration of -COOH groups to 1,638 cm^−1^ (Figure 8A) and 1,635 cm^−1^ (Figure 8B) after adsorption of Pb(II) at pH 3 and 3.5 indicates that the part of -COOH was complexed with the Pb(II), and another part was still present in the form of–COOH (Zhu et al., 2015). The shift of the asymmetric stretching vibration peak of -COO^-^ groups from 1,568 cm^−1^ to 1,540 cm^−1^ indicates most of the -COO^-^ groups were complexed with Pb(II) ions (Figure 8B). When the pH values are higher than 3.5, the shift of the characteristic peaks of the -COO^-^ groups becomes more obvious, which shifted to 1,526 cm^−1^, 1,525 cm^−1^, and 1,523 cm^−1^ after adsorption of Pb(II) at pH 4, 4.5, and 5, respectively (Figures 8C–E). At the same time, the symmetrical stretching vibration peak of -COO^-^ groups also shifts obviously. The obvious shift of adsorption peak proves that the -COO^-^ groups chelates with Pb(II) to form a stable chemical bonding interaction. These results indicate that the stronger chemical complexing action between -COO^-^ groups and Pb(II) mainly contribute to the efficient adsorption of adsorbent towards Pb(II) (Wang et al., 2009) (Scheme 1). The conventional pore adsorption, electrostatic attraction and ion-exchange also have contribution to the adsorption process, but chemical complexing is the dominant driving forces.

### Desorption of Pb(II) and the Regeneration of Adsorbent

**FIGURE 9:**
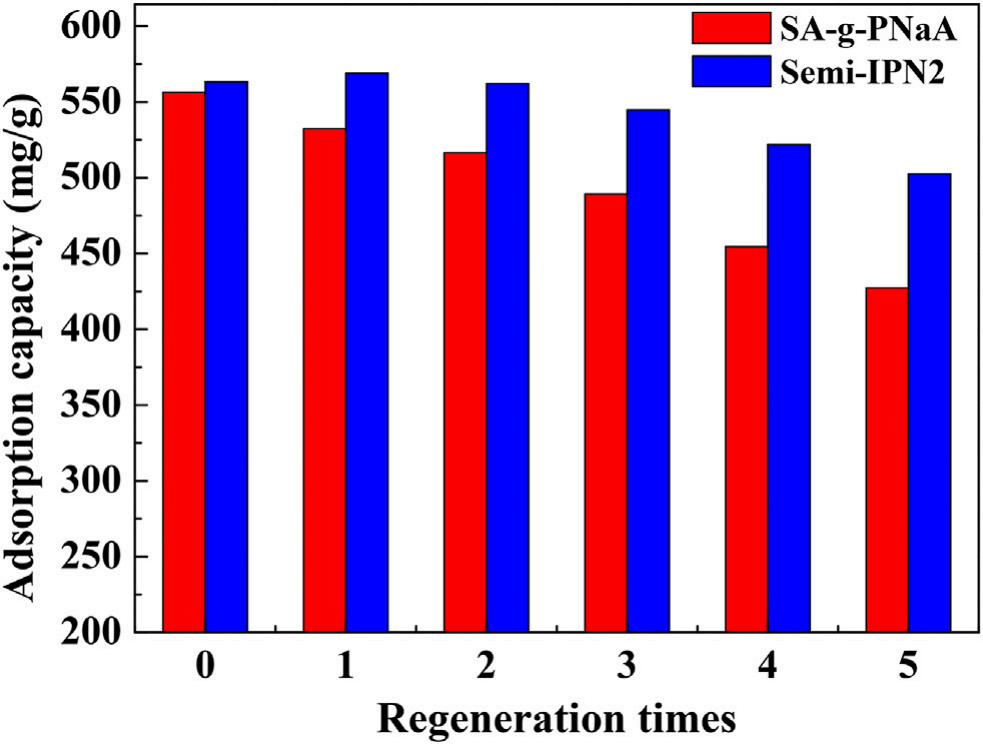
The adsorption capacity of the SA-*g*-PNaA and Semi-IPN2 adsorbents towards Pb(II) after adsorption-desorption for 5 cycles.

An excellent adsorbent usually needs to have excellent regeneration performance and reusability, and it is desired to have the ability to adsorb heavy metals and desorb/recover metal ions at the same time. Regeneration can reduce the use cost of adsorbents, and the recovery of metal ions can reduce the waste of valuable metal resources. With dilute HNO_3_ aqueous solution, the adsorbed metal ions can be quickly and easily desorbed, and the metal ions can be converted into soluble nitrate for reuse at the same time. As shown in Figure 9, the adsorption capacity slightly decreased with increasing the usage times, but the adsorption capacity to Pb(II) still reach 502.5 mg/g (for Semi-IPN2) after reused for 5 cycles, which is obviously higher than that of SA-*g*-PNaA (427.2 mg/g), indicating that the Semi-IPN2 hydrogel adsorbent has relatively better regeneration capability than SA-*g*-PNaA hydrogel. It can be calculated from the desorption data that about 93.6% of the Pb(II) adsorbed by the semi-IPN2 adsorbent can be de-adsorbed and recycled by acid-leaching process, and about 89.9% of the Pb(II) adsorbed by the reused adsorbent (regenerated for 5 times) can be de-adsorbed and recycled. In general, the direct use or reuse of environmentally friendly biopolymer-based hydrogel adsorption materials can simultaneously achieve the purification of heavy metal polluted wastewater, the enrichment of metal ions and the recycling of metal ions, which have great potential as water treatment and separation materials.

## Conclusions

The porous semi-IPN hydrogel adsorbent SA-*g*-PNaA/PVA with very fast adsorption rate and superior adsorption capability towards Pb(II) were prepared by a one-pot polymerization reaction in water medium using PVA as the interpenetrating polymer components. Incorporating 2 wt% of PVA not only improved the adsorption capacity of the hydrogel adsorbent, but also enhanced the gel strength of hydrogel by 119%. The maximum saturation adsorption capacity reaches 784.97 mg/g for the optimal Semi-IPN2 adsorbent, and a high removal ratio of 98.39% was reached at the adsorbent dosage of 2 g/L (initial Pb(II) concentration, 1,150 mg/L). About 93.60% of the adsorbed Pb(II) can be easily desorbed with HNO_3_ and recycled. Incorporating PVA also improved the reusability of hydrogel adsorbent for adsorption of Pb(II), and the Semi-IPN2 adsorbent can still reach the adsorption capacity of 502.5 mg/g toward Pb(II) after regenerated and reused for 5 cycles, which is significantly better than the SA-*g*-PNaA adsorbent. The adsorption kinetic curves fits well with pseudo-first-order kinetic equation at initial stage (*t* ≤ 50 s), which revealed a physical diffusion adsorption process at this stage. However, the adsorption data can be fitted well with pseudo-second-order kinetic equation at second stage (*t* > 60 s) with the best *R* ^2^ value of 1.0000, which reveal that chemical adsorption process is dominant at this stage. The chemical complex and electrostatic attraction between -COO^-^ and Pb(II) are the main driving forces for the high adsorption capacity and fast adsorption rate of the hydrogel, and ion exchange also assists the adsorption process. This paper provides a method for preparing a new type of biopolymer-based environmentally friendly high-efficiency adsorbent. The obtained adsorbent can be used to adsorb and recover Pb(II) from a high-concentration solution, which can be used potentially for high-efficiency purification of heavy metal polluted water and enrichment of metal ion.

## Data Availability

The original contributions presented in the study are included in the article/supplementary material, further inquiries can be directed to the corresponding authors.
